# Multiplexed Illumina sequencing libraries from picogram quantities of DNA

**DOI:** 10.1186/1471-2164-14-466

**Published:** 2013-07-09

**Authors:** Sarah K Bowman, Matthew D Simon, Aimee M Deaton, Michael Tolstorukov, Mark L Borowsky, Robert E Kingston

**Affiliations:** 1 Department of Molecular Biology, Massachusetts General Hospital, and Department of Genetics, Harvard Medical School, Boston, MA 02114, USA; 2 Center for Biomedical Informatics, Harvard Medical School, Boston, MA 02115, USA; 3 Division of Genetics, Brigham and Women’s Hospital, Boston, MA 02115, USA; 4 Present address: Department of Molecular Biophysics and Biochemistry and Chemical Biology Institute, Yale University, West Haven, CT 06516, USA; 5 Present address: Department of Molecular Biology, Massachusetts General Hospital, Boston, MA 02114, USA; 6 Present address: Novartis Institutes for BioMedical Research, Cambridge, MA 02139, USA

**Keywords:** Illumina, ChIP-seq, Multiplex, Barcoding, Library preparation

## Abstract

**Background:**

High throughput sequencing is frequently used to discover the location of regulatory interactions on chromatin. However, techniques that enrich DNA where regulatory activity takes place, such as chromatin immunoprecipitation (ChIP), often yield less DNA than optimal for sequencing library preparation. Existing protocols for picogram-scale libraries require concomitant fragmentation of DNA, pre-amplification, or long overnight steps.

**Results:**

We report a simple and fast library construction method that produces libraries from sub-nanogram quantities of DNA. This protocol yields conventional libraries with barcodes suitable for multiplexed sample analysis on the Illumina platform. We demonstrate the utility of this method by constructing a ChIP-seq library from 100 pg of ChIP DNA that demonstrates equivalent genomic coverage of target regions to a library produced from a larger scale experiment.

**Conclusions:**

Application of this method allows whole genome studies from samples where material or yields are limiting.

## Background

As the price of high throughput sequencing declines, it is easier for researchers to apply genome-wide approaches to diverse samples of DNA. One particularly interesting type of sample is DNA enriched from techniques that map regulatory interactions on chromatin. These techniques include chromatin immunoprecipitation (ChIP), which purifies fragments of DNA that bind to a regulatory protein, such as a transcription factor or a covalently modified histone. When ChIP is applied to limited cell numbers, such as rare cell populations or a specific cell type that is difficult to harvest, the amount of recovered DNA is frequently limiting for sequencing library production. Multiplex library protocols typically require several nanograms or microgram amounts of input DNA, while ChIP from limited cell numbers, such as 10^5^*Drosophila* cells or 10^4^ mammalian cells, can yield far less.

The problem presented by limited amounts of input DNA has been addressed in different ways. One strategy is to make multiple copies of the purified DNA fragments prior to library production, either by PCR [1,2] or by in vitro transcription [3]. This increases the amount of the input DNA to the microgram range, so it is amenable to sequencing library construction. However, additional amplification cycles can skew sequencing results [4], and these methods are inherently time-consuming, involving several additional enzymatic steps. Other strategies for library construction from small amounts of DNA are not suitable for ChIP analysis because they require unfragmented genomic DNA as input material [5-7].

**Figure 1:**
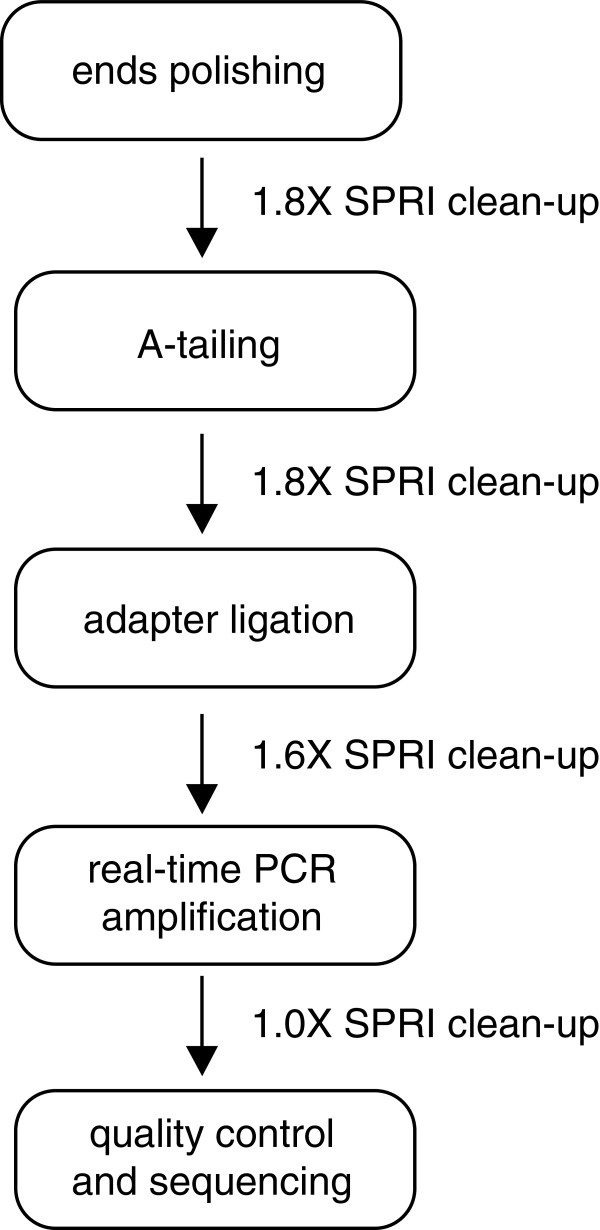
**Workflow.** The ratio of the volume of suspended SPRI beads to the volume of sample is indicated.

To avoid these drawbacks, we developed a simple and fast library construction protocol (Figure 1) that uses sub-nanogram quantities of fragmented DNA as input, and avoids pre-amplification and overnight steps. The resulting libraries are barcoded and suitable for multiplexed analysis on the Illumina platform. The oligo design is based on the Illumina TruSeq sample preparation and the protocol draws from that method, as well as others [8,9] that require nanograms to micrograms of input material. The advantage of the protocol reported here is that it allows library construction from 100 pg of ChIP DNA using a customizable, kit-independent workflow.

## Results and discussion

Illumina DNA library construction consists of four major steps: end polishing, A-tailing, adapter ligation, and library amplification. Between steps, enzymatic reactions are purified using solid phase reversible immobilization beads (SPRI beads). To adjust these steps for use with picograms of input, we introduced modifications that are outlined in Table 1.

**Table 1:** Comparison of Illumina and modified method

**Parameter**	**Illumina method**	**Drawback**	**Modified method**	**Benefit**
Adapters	Indexed	Entire sample receives one barcode	Universal	Flexibility; multiple barcodes can be added later
Size selection	Gel-mediated	Time-consuming; sample loss	No size selection	Time savings; sample retention
Amplification	10-18 cycles	Potentially under-optimized	Monitored by qPCR	Stop cycling during log growth phase
All steps	Constrained by kit	Difficult to modify	Transparent and kit-independent	Flexibility

**Figure 2:**
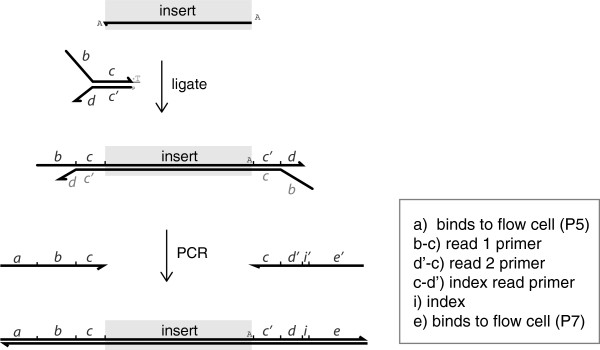
**Oligonucleotide design and products of protocol.** P5 and P7 are names given by Illumina to the oligo sequences that bind to the flow cell.

We designed universal adapters and barcoded amplification oligos that would be compatible with single- or paired-end sequencing on the Illumina platform. The Illumina multiplex protocol for DNA introduces the barcode (or “index”) to the library in the adapter oligo. We preferred to use universal adapter sequences and add the barcodes during the amplification phase, a strategy used by others [10,11] and also developed into a DNA library prep kit (NEBNext) sold by New England Biolabs. Use of universal adapters and indexed amplification primers offers the option to save part of the adapter-ligated DNA sample and, if experimentally necessary, amplify a library with an alternative barcode. We designed universal adapter oligos with similar melting temperatures to those developed by Illumina for paired-end sequencing, and included sites of phosphorylation and phosphorothioate linkages [12]. Ligation of universal adapters to DNA fragments creates products that are extended by PCR to produce barcoded samples containing the identical sequences used for Illumina TruSeq multiplexing (see Additional file 1; also TruSeq DNA Sample Preparation Guide, Part No. 15005180 Rev. A). These oligos produce libraries that are compatible with conventional data analysis pipelines (Figure 2).

Additional modifications to the Illumina protocol include skipping the gel-mediated size selection step and monitoring the amplification of the library by quantitative PCR (qPCR). Illumina recommends purifying the ligation products on a gel to remove excess adapters. By adding less than 1 uM adapters to the ligation reaction, we generally avoid excess adapters and find that gel purification can be avoided for samples fragmented either by enzymes (this study) or sonication [13]. Following adapter ligation, library amplification both enriches for DNA fragments with an adapter ligated to both ends and increases the amount of DNA in the library. Illumina protocols recommend 10 cycles of PCR when starting with one microgram of input DNA, and 18 cycles when starting with 5 nanograms, but it is difficult to know a priori how to optimize cycle number for alternative sample amounts. Following the amplification in real time by monitoring SYBR Green fluorescence allows the reaction to be stopped during the exponential phase and before the reaction plateaus. This allows the maximum amount of DNA to be produced for each library while preventing over-cycling and heteroduplex formation, which can interfere with downstream quantitation [11,14]. While we have not noticed any obvious decrease in the sequencing data quality when SYBR Green is included in the PCR step, one alternative to avoid this would be to amplify half the adapter-ligated DNA using real-time PCR to determine cycle number, and repeat the reaction without SYBR Green using the remaining sample. (For a detailed protocol, please see Additional file 2).

We applied the modified library construction protocol to approximately 100 pg of DNA from *Drosophila* embryos enriched by ChIP against trimethylated lysine 27 of histone H3 (H3K27me3). H3K27me3 is a repressive histone modification found in broad domains throughout the *Drosophila* genome, notably at the Bithorax Complex (BX-C), a 300 kb region containing 3 homeotic genes: *Ubx*, *Abd-A*, and *Abd-B*. We reasoned that successful ChIP-seq library construction from picograms of DNA would enrich the same domains as libraries constructed in a larger scale format.

**Figure 3:**
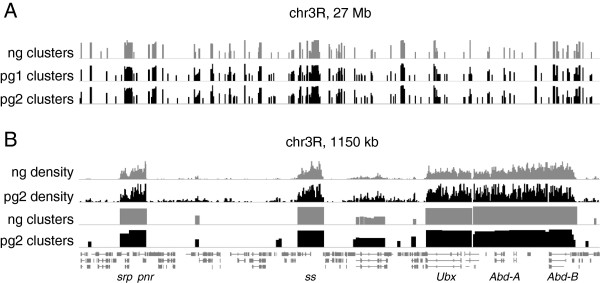
**The modified library protocol recapitulates known regions of enrichment in H3K27me3 ChIP.** Qualitative depiction of tag density and cluster enrichment. ng: data generated from 5–50 ng ChIP DNA by modENCODE. pg1, pg2: biological replicates of data generated from ~100 pg ChIP DNA for this study. The chromatin in the ng experiment is fragmented by sonication, while the chromatin in the pg experiment is fragmented by micrococcal nuclease. **A**: Clusters of region of enrichment on the entirety of chr3R with a significance threshold of Z-score =3 and enrichment of 2-fold or more. y-axis is 0–3 for all samples. **B**: Input subtracted tag density and corresponding regions of enrichment (based on M-values) in ~1000 kb of chr3R. Y-axis is 0–250 for tag density samples and 0–3 for cluster samples. chr3R: chromosome 3R. Selected genes noted in italics.

**Figure 4:**
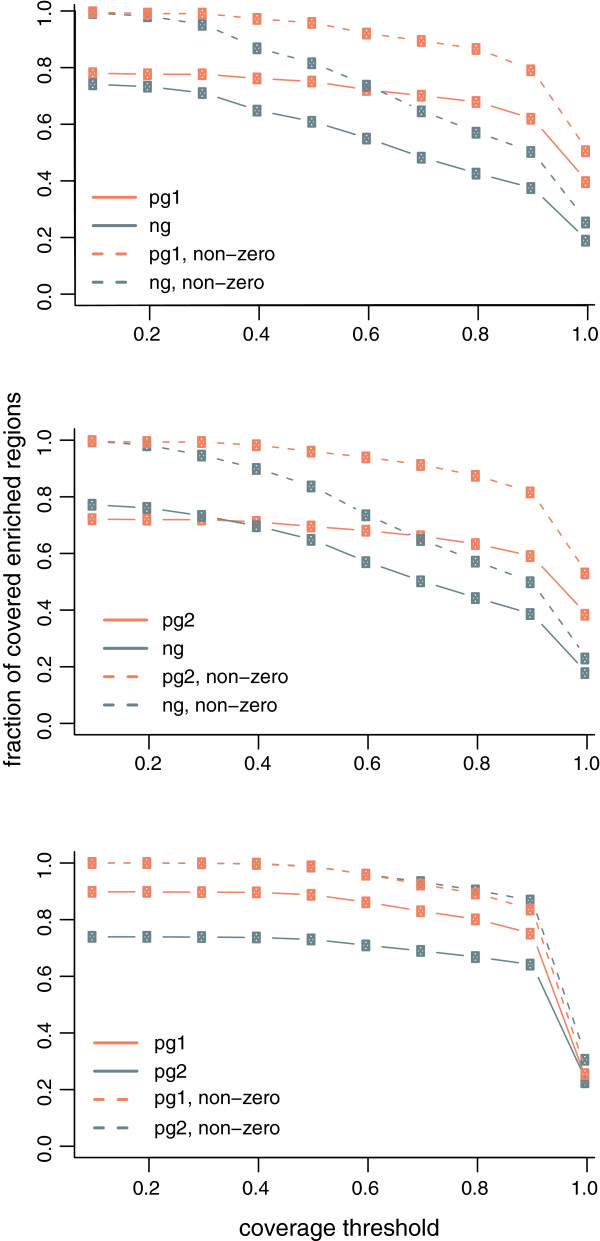
**Pairwise comparison of regions of enrichment in nanogram- and picogram-scale libraries.** Each panel depicts a single pairwise comparison: ng and pg1, ng and pg2, and pg1 and pg2. Coverage of each region of enrichment in one sample (color-coded in the plots) by the regions of enrichment identified in another sample was computed as described in Methods. Then, the fractions of the total number of the regions of enrichment with coverage above the specified threshold were computed and presented in plots (solid lines). Since some regions of enrichment are not reproduced at all in different libraries, the same fractions were computed for the regions that have non-zero coverage (dashed lines) to address possible bias in the analysis.

The modified protocol produces data with enriched regions qualitatively and quantitatively similar to data from a related, larger scale experiment (Table 2). Multiple independent ChIP experiments from 10^5^ cells reproducibly yielded measurements of 50–150 pg of DNA when assayed with PicoGreen reagent. Library preparation was performed on the output of two of these ChIP experiments, followed by single-end sequencing. Tag density across the *Drosophila* genome from the picogram-scale libraries was compared to tag density from a similar experiment performed at larger scale by the modENCODE consortium (5–50 ng input) [15]. Regions of enrichment were also identified. While the biological samples and chromatin fragmentation were not identical (see Methods), we found enrichment of similar genomic regions at multiple levels of scale (Figure 3). Furthermore, a genome-wide assessment demonstrates strong overlap between enriched regions in the picogram- and nanogram-scale experiments, and good reproducibility of the results from the picogram samples (Figure 4). This shows that using the method described here, it is possible to produce ChIP-seq results similar to larger scale experiments by using only ~100 pg of DNA as input for library preparation.

**Table 2:** Data for sequence reads and called clusters of enrichment

	**Total reads**	**Filtered, aligned reads**	**Clusters**
**nanogram input**	5,744,566	5,385,609 (93%)	n/a
**nanogram ChIP**	7,891,078	7,612,895 (96%)	723
**picogram1 input**	52,393,508	46,056,072 (88%)	n/a
**picogram1 ChIP**	23,305,231	15,996,046 (69%)	1050
**picogram2 input**	28,246,963	25,223,327 (89%)	n/a
**picogram2 ChIP**	20,922,935	10,783,494 (52%)	1285

## Conclusions

This picogram-scale protocol should be broadly useful not only for small scale ChIP experiments, but for any high throughput sequencing experiment where material or yields are limiting and multiplexed sample analysis is desired. We have yet to apply this protocol to ChIP-enriched DNA from mammalian cells. Since ChIP from 5,000 to 10,000 mammalian cells enriches 10–50 pg of DNA and yields adequate depth of sequencing by other library construction methods [1,3], it is reasonable to anticipate that this picogram-scale library construction protocol may also prove useful for experiments in genomes larger than that of *Drosophila*.

Several steps may serve as variables that can tailor the protocol to different DNA samples or quantities. For instance, by monitoring library amplification in real time, cycle number can be kept to a minimum while still ensuring that the reaction has reached the exponential phase and produced enough DNA for the final library. Another variable is the amount of adapter added in the ligation reaction. This may be titrated up or down to accommodate different sample amounts. Two reports demonstrated that use of alternative polymerases or even different thermocyclers can enhance library preparation from specific types of DNA, such as samples unusually low or high in GC percentage, or ancient DNA [4,14]. Finally, the oligo design used in this protocol is transparent and allows all samples to be ligated to universal adapters, while the choice of barcode is delayed until the library amplification step. With simple adjustments to steps in this protocol, it may be customized to a wide array of DNA input sources and concentrations. This should prove useful for high throughput sequencing from small or rare samples of cells, or from DNA enrichment techniques that are particularly low-yielding.

## Methods

### End preparation and adapter ligation

End polishing reactions (50 uL) contained 1X T4 ligase buffer (NEB, Ipswich, MA, USA), 0.4 mM dNTPs, 7.5 U T4 polymerase (NEB), 2.5 U Klenow polymerase (NEB), 25 U polynucleotide kinase and were incubated for 30 minutes at 20C in a thermocycler. SPRI cleanup was performed with 1.8X beads ratio (90 uL beads suspension) as described below, and eluted with 16.5 uL water. A-tailing reactions (25 uL) contained 16 uL sample, 1X NEB buffer 2, 0.2 mM dATP, 7.5U Klenow 3’-5’ exo minus (NEB) and were incubated for 30 minutes at 37C. SPRI cleanup was performed with 1.8X beads ratio (45 uL beads suspension) and eluted with 9.5 uL of water. Adapter ligation reactions (25 uL) contained 9 uL sample, 1X rapid T4 ligase buffer (Enzymatics, Beverly, MA, USA), 0.01 uM annealed universal adapter, 150U T4 rapid ligase (Enzymatics), and were incubated for 15 min at room temperature. SPRI cleanup was performed with 1.6X beads ratio (40 uL beads suspension) and eluted with 10.5 uL water.

### SPRI sample clean-up

SPRI beads (Agencourt AMPure XP, Beckman Coulter) were brought to room temperature before use. Beads in suspension were added to DNA sample in low retention microfuge tubes and mixed by pipetting. The sample was incubated at room temperature for 5 minutes outside a magnetic rack, and 8 minutes inside a magnetic rack. While keeping the tube in the rack, supernatant was removed by aspiration, and the beads pellet was washed twice for 30 seconds with 200 uL of 80% ethanol (freshly prepared), taking care not to disturb the pellet by addition of the wash. Complete removal of the second wash was sometimes assisted by centrifuging the tubes briefly and replacing them in the magnetic rack. The pellets were allowed to dry at room temperature in the magnetic rack for 5 minutes with open caps. The beads pellet was resuspended in the required volume of water by pipetting, allowed to incubate outside the magnetic rack for one minute, inside the magnetic rack for one minute, and the eluate removed from the beads by pipette and used for the next step. A cost-effective alternative to purchasing AMPure beads is making them in the lab using paramagnetic carboxyl-coated beads in PEG/NaCl buffer [16].

### Library amplification by qPCR

PCR reactions (50 uL) consisted of 1X Phusion HF master mix (NEB), 0.2 uM universal primer, 0.2 uM barcoded primer, 1X SYBR Green I (Invitrogen), and 0.5 uL Rox (USB). Thermocycling was performed by initially denaturing for 30 seconds at 98C; then multiple cycles of the following: 10 seconds denaturation at 98C, 20 seconds annealing at 64C, and 45 seconds extension at 72C. Reactions were stopped at the end of the extension, after SYBR green reported reaction kinetics in the log phase for several cycles. The thermocycler used in these experiments was an Applied Biosystems 7500 Fast Real-Time PCR System.

### Illumina sequencing and data analysis

Libraries were diluted and pooled for cluster generation and sequence analysis on one lane of an Illumina HiSeq2000 by a local NGS service provider, who sequenced the library using standard manufacturer’s procedures. Sequenced tags were aligned to the *D. melanogaster* genome (dm3) using Bowtie aligner [17]. Only tags with no more than two mismatches in the first 28 bp of the tag were retained. Tags with up to five alignments were accepted to allow interrogation of repetitive regions and, in the case of tags with multiple mappings, only the best alignment was reported and taken for further analysis in the case of tags with multiple mappings. In the picogram samples, reads mapping to the same genomic positions constituted a higher proportion than in the nanogram sample. However, a plurality of the profiled genomic coordinates are associated with a single read count (at least 40%; data not shown). The genomic distributions of mapped tags were analyzed using SPP package [18]. In short, positions in the genome with the numbers of mapped tags above the significance threshold defined by a Z-score of seven were identified as anomalous, potentially resulting from amplification bias. The tags mapped to such positions were discarded. Since the positions of sequenced tags correspond to 5’-ends of the DNA fragments, these positions were shifted by the half of the average fragment size (75 bp) towards the fragment 3’-ends to represent centers of the DNA fragments. The positions of tags mapping to positive and negative DNA strands were combined. Tag density profiles (Figure 3) along chromosomal coordinates were calculated for each sample using Gaussian kernel with 50-bp bandwidth. Continuous regions of enrichment (Figure 3B) were identified with SPP package using default parameters. Only regions meeting the significance threshold of Z-score=3 and with enrichment 2-fold and more were retained for further analysis. The positional overlap between enriched regions (Figure 4) was identified in pair-wise comparison of the samples. As a measure of reproducibility of the H3K27me3 enrichment, the coverage value was computed for each region as a fraction of base pairs belonging to this region and to any other region in another sample. The fraction of enriched regions having coverage values above the specified threshold was identified to analyze reproducibility between the samples. Since presence of a considerable fraction of enriched regions that are called in one sample and not called in another sample can obscure the analysis, we computed the fractions of the reproduced enriched regions both for all regions and for the regions that have non-zero coverage. Results from a comparison to randomized regions are provided for reference (Additional file 3) and illustrate the significance of the enrichment overlap observed in the real data.

### Availability of supporting data

The datasets supporting the results of this article are available in NCBI’s gene Expression Omnibus and are accessible through GEO Series accession number GSE48431 (http://www.ncbi.nlm.nih.gov/geo/query/acc.cgi?acc=GSE48431).

## Abbreviations

ChIP: Chromatin immunoprecipitation; ChIP-seq: ChIP followed by high throughput sequencing; H3K27me3: Trimethylated lysine 27 of histone H3; chr3R: Chromosome 3R; BX-C: Bithorax complex; Ubx: Ultrabithorax; Abd-A: Abdominal-A; Abd-B: Abdominal B.

## Competing interests

The authors declare no competing financial or non-financial interests.

## Authors’ contributions

SKB and MDS developed the library protocol. MDS and MLB designed the oligos. AMD did the ChIP experiment, and MT analyzed the sequencing data. The manuscript was written by SKB with contributions from MT and MDS. REK participated in the design and coordination of the study and helped to draft the manuscript. All authors read and approved the final manuscript.

## Supplementary Material

Additional file 1 List of the oligonucleotide sequences used for library construction. Click here for file

Additional file 2 Detailed protocol of library construction. Click here for file

Additional file 3: Figure S1 Pairwise comparison of the randomized data. Click here for file
