# Supplementary material for: Multiplexed Illumina sequencing libraries from picogram quantities of DNA
Source: BMC Genomics. 2013 Jul 9;14:466. doi: 10.1186/1471-2164-14-466 (PMC3711846; doi:10.1186/1471-2164-14-466)
Supplement: Additional file 1 — List of the oligonucleotide sequences used for library construction. [file 1471-2164-14-466-S1.pdf]

**Oligo sequences**

Oligonucleotides were synthesized by Integrated DNA Technologies with phosphorothioate (*) linkages or 5' phosphate (Phos) as indicated, and HPLC purified. Index/barcode sequence is in **bold**. Oligo design is based on sequences from Illumina: Oligonucleotide sequences © 2007-2011 Illumina, Inc. All rights reserved. Derivative works created by Illumina customers are authorized for use with Illumina instruments and products only. All other uses are strictly prohibited.

Universal adapters:

A.U1:
5'-ACACTCTTTCCCTACACGACGCTCTTCCGATC*T-3'

A.U2:
5'-Phos/GATCGGAAGAGCACACGTCTGAACTCCAGTCAC-3'

Index primers:

PCR.1:
5'-CAAGCAGAAGACGGCATACGAGAT**CGTGAT**GTGACTGGAGTTCAGACGTGTGCTCTTCCGATC*T-3'

PCR.2:
5'-CAAGCAGAAGACGGCATACGAGAT**ACATCG**GTGACTGGAGTTCAGACGTGTGCTCTTCCGATC*T-3'

PCR.3:
5'-CAAGCAGAAGACGGCATACGAGAT**GCCTAA**GTGACTGGAGTTCAGACGTGTGCTCTTCCGATC*T-3'

PCR.4
5'-CAAGCAGAAGACGGCATACGAGAT**TGGTCA**GTGACTGGAGTTCAGACGTGTGCTCTTCCGATC*T-3'

PCR.5
5'-CAAGCAGAAGACGGCATACGAGAT**CACTGT**GTGACTGGAGTTCAGACGTGTGCTCTTCCGATC*T-3'

PCR.6
5'-CAAGCAGAAGACGGCATACGAGAT**ATTGGC**GTGACTGGAGTTCAGACGTGTGCTCTTCCGATC*T-3'

Universal primer:

PCR.U
5'-AATGATACGGCGACCACCGAGATCTACACTCTTTCCCTACACGACGCTCTTCCGATC*T-3'
