# Supplementary material for: Multiplexed Illumina sequencing libraries from picogram quantities of DNA
Source: BMC Genomics. 2013 Jul 9;14:466. doi: 10.1186/1471-2164-14-466 (PMC3711846; doi:10.1186/1471-2164-14-466)
Supplement: Additional file 2 — Detailed protocol of library construction. [file 1471-2164-14-466-S2.pdf]

<u>Picogram-scale library construction</u>
Bowman et al., 2012

<u>1. End repair: 50 uL volume</u>

37.5 uL	sample +H2O
2 uL	10 mM dNTPs
5 uL	NEB T4 ligase buffer
2.5 uL	NEB T4 polymerase
0.5 uL	NEB Klenow polymerase
2.5 uL	NEB T4 PNK

incubate 30 min at 20C in thermocycler
SPRI cleanup (see below), 1.8X beads ratio (90 uL beads for 50 uL reaction)
Elute with 16.5 uL water

<u>2. A-tailing: 25 uL volume</u>

16 uL	sample
2.5	NEB buffer #2
5 uL	1 mM dATP
1.5 uL	NEB Klenow 3'-5' exo minus

incubate 30 min at 37C
SPRI cleanup, 1.8X beads ratio (45 uL beads for 25 uL reaction)
Elute with 9.5 uL of water

<u>3. Ligate adapters: 25 uL volume</u>

9 uL	sample
12.5 uL	Enzymatics 2X rapid T4 ligase buffer
0.25 uL	1 uM universal adapters
2.5 uL	Enzymatics T4 rapid ligase

optional: include a "no insert" reaction with 9 uL water instead of DNA
incubate 15 min at room temperature
SPRI cleanup, 1.6X beads ratio (40 uL beads for 25 uL reaction)
Elute in 10.5 uL water

<u>4. qPCR to determine appropriate cycle number and amplify library</u>

25 uL	Phusion HF master mix
1 uL	universal primer 10 uM
1 uL	index primer 10 uM
1 uL	1:2000 SYBR Green
0.5 uL	Rox
10 uL	adapter ligated DNA
9.5 uL	H2O

cycling: 98C 30 sec; 98C 10 sec, 64C 20 sec, 72C 45 sec

Monitor reaction as it happens; stop immediately following extension step at a cycle number that yields the most DNA while still in the logarithmic phase of the reaction (ideally, this would be right before the reaction begins to plateau because of reagent consumption). If the "no insert sample" is included from step 4, the amplification kinetics of the ChIP DNA can be compared to amplification kinetics of adapters alone. Successful ChIP DNA library preparation often (but not always) correlates with earlier entry into visible log-phase amplification than the no-insert sample.

In a successful ChIP DNA library, stringent SPRI purification after the amplification step (1.0X beads ratio; or 50 uL beads for the 50 uL PCR reaction) and elution in a volume of 10 uL or less usually removes the majority of adapter/primer dimers (120 bp) from the rest of the library (often 250 bp and above) and obtains a library concentration of 10 nM or more.

SPRI cleanup:

1. Warm SPRI beads to room temp
2. Add beads to reaction
3. Mix by pipetting up and down; incubate at room temp 5 min
4. Capture beads on magnetic stand, 8 min
5. Remove supernatant and discard
6. Add 200 uL of freshly made 80% ethanol, without disturbing pellet, rinse 30 sec.
7. Do step 6 again; aspirate all ethanol (quick spin if necessary)
8. Dry pellet 5 minutes (tubes sit in magnetic rack with cap open)
9. Take pellet in desired volume of water to elute: pipette up and down to resuspend pellet, incubate 1 min in suspension, capture 1 min, remove elution to new tube.
