# Supplementary material for: Multiplexed Illumina sequencing libraries from picogram quantities of DNA
Source: BMC Genomics. 2013 Jul 9;14:466. doi: 10.1186/1471-2164-14-466 (PMC3711846; doi:10.1186/1471-2164-14-466)

Bowman et al. Figure S1: Pairwise comparison of the randomized data.
The regions of enrichment identified in the samples under comparison were shuffled within each chromosome preserving the region lengths. After the shuffling, the same approach was used for the comparison as that applied to the real data. In short, the coverage of the regions of enrichment in one sample by the regions of enrichment from another sample was identified, and the fractions of the regions with the overlap above the thresholds indicated on x-axis were computed (see Methods for detail). The plots are shown for the fractions of the total number of regions of enrichment (solid lines) and for the fractions of the number of regions that have non-zero coverage (dashed lines).

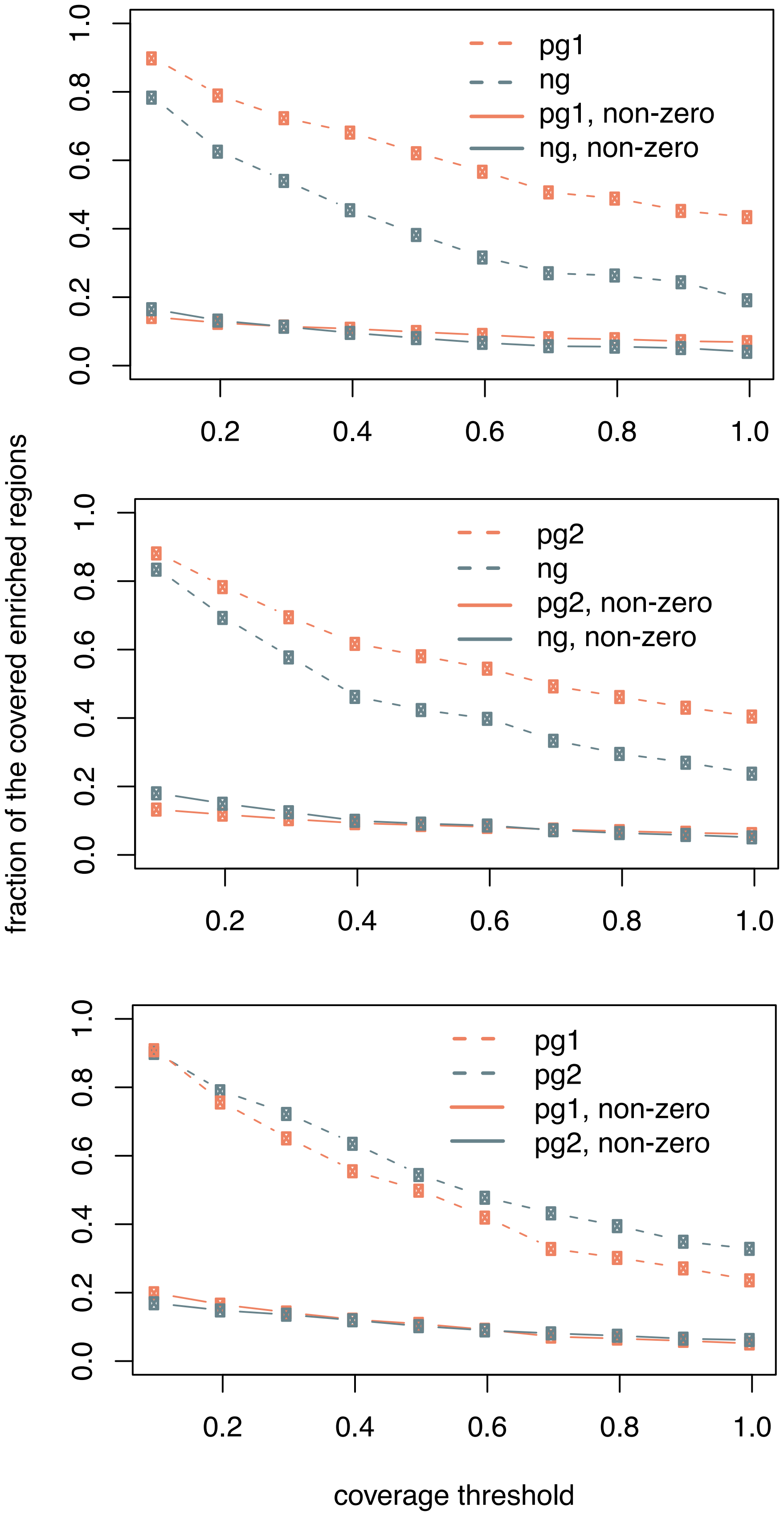

Supplement: Additional file 3: Figure S1 — Pairwise comparison of the randomized data. [file 1471-2164-14-466-S3.pdf]
